# Nurses’ knowledge, perceived barriers, and practices regarding cancer pain management: a cross-sectional study from Palestine

**DOI:** 10.1186/s12909-019-1613-z

**Published:** 2019-05-23

**Authors:** Haneen A. Toba, Ahmad M. Samara, Sa’ed H. Zyoud

**Affiliations:** 1 Department of Medicine, College of Medicine and Health Sciences, An-Najah National University, Nablus, 44839 Palestine; 2 Poison Control and Drug Information Center (PCDIC), College of Medicine and Health Sciences, An-Najah National University, Nablus, 44839 Palestine; 3 Department of Clinical and Community Pharmacy, College of Medicine and Health Sciences, An-Najah National University, Nablus, 44839 Palestine

**Keywords:** Cancer pain, Nurses, Knowledge, Practices, Perceived barriers, Palestine

## Abstract

**Background:**

Accurate knowledge and good pain evaluation and documentation practices should be present for efficient pain management. In this study, we aimed to assess the knowledge and practices of nurses relating to the management of cancer pain in Palestine, and to determine the barriers to efficient pain control in cancer patients.

**Methods:**

A cross-sectional survey took place at 8 hospitals across Northern West Bank. A convenience sample of 220 Nurses working in governmental and private hospitals in West Bank/Palestine was studied. For that purpose, a questionnaire was developed to assess knowledge, practices, perceived barriers, and delaying processes relating to cancer pain management (CPM).

**Results:**

In total, 220 questionnaires were completed with a response rate of 88%. Participants’ mean age was 30.34 years. Overall, 69.5% worked in governmental hospitals, 26.8% worked in the private sector and the remainder worked in both governmental and private sectors. The correct response rate to questions that assess knowledge relating to cancer pain control was calculated and a mean knowledge score was found to be 5.1 with a standard deviation of 2.1. A relationship between the knowledge score and the sample characteristics was made and showed that males scored significantly higher (*p* = 0.001) than females with median scores of 6 [4–7] and 5 [3–6] for males and females, respectively. Inadequate pain assessment (76.8%), insufficient knowledge of pain control (70.5%) and strict regulation on opioid use (69.5%) were the most frequently perceived barriers. Nurses reported that they would assess pain on every round and check all items related to pain assessment. Contacting the physician for the prescription of opioids was cited as the main delaying process by 56.4% of participants.

**Conclusions:**

This study allowed us to recognise the knowledge deficit and the barriers to effective management. On the other hand, the analysis has shown good pain documentation practices among nurses. Those knowledge deficits demonstrate the need for more education about CPM. The improvement of coordination and communication between physicians and nurses seems to play a crucial role in CPM, as contacting physicians was cited as the most delaying process in CPM by nurses.

**Electronic supplementary material:**

The online version of this article (10.1186/s12909-019-1613-z) contains supplementary material, which is available to authorized users.

## Background

Cancer patients will experience pain at some time or other. This pain can be due to the cancer’s direct damage to the body or from the treatment. Overall, 92.5% of 174 cancer patients who were admitted to a pain management unit suffered from pain that was described as more than moderate despite previous treatment [1]. The low pain control quality was attributed to the under-dosage of medications, insufficient intake scheduling, and hesitancy towards using a strong opioid [1, 2].

A Jordanian study including 200 Jordanian nurses has shown that these nurses have knowledge gaps concerning pain management, which negatively impact the quality of pain control in cancer patients [3]. Nurses’ knowledge about opioids’ role in pain management was also reported as low by a study conducted in Italy, highlighting their irrational fear of the opioids’ potential to result in addiction or respiration inhibition [4]. In Korea, another study demonstrated poor knowledge and improper practices among physicians and nurses [5].

Barriers to the adequate management of cancer pain according to an Israeli study were the inadequate assessment of pain and outcomes of treatment, insufficient knowledge among health professionals, physicians’ poor attitude towards prescribing opioids, and reluctance to take opioids by patients. Interestingly, nurses’ reluctance to the administration of opioids and their concerns relating to excessive regulation were not cited as common barriers to adequate pain control [6].

In Palestine, cancer is reported as the second leading cause of death (14.2%), with cases usually being diagnosed in a late stage, leading to a greater load on patients and healthcare staff, and a poorer quality of life for patients [7], which was found to be low among Palestinian cancer patients [8]. Additionally, nurses play an essential role in cancer pain management but nursing education curriculum remain lacking in basic pain management principles [9]. To the best of our knowledge, cancer pain management (CPM), in terms of knowledge, practices and perceived barriers among Palestinian nurses, has never been targeted by research. The aim of our study was to demonstrate how each of these groups of obstacles contributes to the problem of cancer-related pain management in Palestine and the need to improve clinical practices and education, as well as related policies. In the current study, we described the knowledge and practices of nurses relating to the management of cancer pain in Palestine and highlighted some barriers that could prevent the adequate control of pain among cancer patients. By evaluating the knowledge, practices and perceived barriers relating to the management of cancer-related pain among nurses working with cancer patients, it will be easier to identify and develop educational resources and training programs for palliative care; it will also emphasise the importance of communication and consultation between healthcare professionals.

## Methods

### Study design

The study was conducted using a cross-sectional survey design.

### Study setting and participants

This study took place between May and September 2017 at 8 hospitals (7 governmental and 1 private) in Northern West Bank. Nurses responsible for the care of cancer patients were eligible to participate. Institutional Review Board approval was granted by 8 participating hospitals (Al Watani/Nablus, Tubas (Turkish), Jenin (Khalil Suliman), Tulkarm (Thabit Thabit)), Salfit (Yasser Arafat), Nablus (Rafidia), Qalqiliya (Darwish Nazal) and An-Najah National University Hospital).

### Sample size and sampling technique

The estimated number of nurses licensed by the Palestinian Nursing Association who worked at the surveyed hospitals and were responsible for cancer patients in their practice was around 500 [10]. Accordingly, the sample size of 218 was calculated by using Raosoft sample size calculator [11], after setting the indicator percentage at 0.50, the margin of error at 5%, and the confidence interval at 95%. A convenience sample of 250 nurses in governmental and private hospitals in West Bank/Palestine was identified during the study period. In order to minimise erroneous results and to avoid dropout, we increased the target sample size to 250 nurses.

### Inclusion and exclusion criteria

Inclusion criteria demands that the hospital in which the participant works admits and treats cancer patients, and that the participant is an active part of the treatment process for such patients. The exclusion criteria, on the other hand, rule out those who are not allowed to make decisions or write orders in the management of cancer patients such as students and those in training. After collecting the data, those who did not complete all items of the questionnaire were also excluded.

### Instruments and measures

A questionnaire was developed based on tools that were used in previous similar studies in other countries [4–6, 12–27]. The questionnaire consisted of five main sections. The first contained the demographic data of the participants, in addition to some professional questions such as the nature and duration of their interaction with cancer patients during their work time.

The second section included a set of 14 true or false questions to test the participants’ knowledge [5] regarding the principles of managing cancer pain and differentiating it from other causes of pain, analgesic drug properties, non-pharmacological pain management, calculating opioid dose and routes of administration, the proper timing of assessment after administering the opioid, safety profile of opioid analgesics, tolerance development to opioids and the prevalence of refractory cancer pain. Subsequently, a knowledge score was calculated by allocating one point for each correctly answered question of the 14 knowledge questions and adding these points up. Higher knowledge score means better knowledge. Permission to use the questionnaire tool for this study was obtained from Professor Yeol Kim via personal email.

In addition, statements of agreement questions were used in the third and fourth sections. The third section was regarding the perceived barriers in dealing with cancer patients. Questions about perceived barriers were separated into three groups: patient-, medical staff-, and health-care system-related barriers. The forth section included questions regarding pain assessment and documentation practices and enquired about the occasions of pain assessment in cancer patients, aspects of pain assessed, and whether nurses documented pain after every assessment practice in the patient file.

The last section investigated the perceptions regarding the most delaying process in opioid administration. Four options were listed for the participants to choose from: administration of opioid to the patient, getting to the pharmacy to obtain the opioid, contacting the physician for an opioid prescription, or the delaying process is difficult to recognize.

The questionnaire was developed initially in English, and then translated into Arabic before being offered to the participants (see Additional file 1: English and Arabic version of the instrument). Face and content validity of the final questionnaire was discussed and judged by three expert’s academicians in the related field for assessing the completeness, appropriateness, meaning of terms, logical sequence of the statements, and organization and the accuracy. Some questions were modified as recommended. The developed questionnaire was initially piloted among fifteen nurses before the start of the study to evaluate clarity, and time considerations; only minor modifications were needed. Internal consistency reliability for knowledge scale was good, with Cronbach’s α = 0.771.

### Data analysis

Descriptive analyses were implemented to describe the characteristics of participants and their answers to questions related to knowledge, practices and the perception of barriers. The relationship between knowledge score and participants characteristics was also obtained. Statistical analyses were performed using SPSS version 21.0. Statistics that present the data include frequencies and percentages, mean ± standard deviation (SD), or median with interquartile range as appropriate. Data were analysed using the Kruskal-Wallis and Mann-Whitney U tests at *p* < 0.05. Internal consistency reliability for knowledge scale was tested via Cronbach’s alpha coefficient.

## Results

### Sample characteristics

In total, 250 questionnaires were distributed and 220 were completed with a response rate of 88%. They had a mean age of 30.34 years (SD = 8.1), with a mean experience in cancer patient treatment of 5 years. Male participants comprised 47.7% of all respondents. Palestine was the country of education for most of the participants (94.5%), with 69.5% working in governmental hospitals, 26.8% working in the private sector and the remainder working in both governmental and private sectors. Table 1 shows the demographic data and characteristics of participants.

**Table 1:** Relationship between knowledge score and characteristics of participants

Characteristics	Number of nurses (%)	Knowledge score^a^ Median [interquartile]	*P* value^b^
Age (year)			
Less than 40	183 (83.2)	5.0 [4.0–7.0]	0.926^c^
40 or more	37 (16.8)	5.0 [4.0–7.0]	
Gender			
Male	105 (47.7)	6.0 [4.0–7.0]	
Female	115 (52.3)	5.0 [3.0–6.0]	**0.001** ^**c**^
Country of education			
Palestine	208 (94.5)	5.0 [4.0–7.0]	0.058^c^
Abroad	12 (5.5)	6.0 [5.3–7.0]	
Type of work			
Governmental	153 (69.5)	5.0 [4.0–7.0]	0.633^d^
Private sector	59 (26.8)	5.0 [4.0–7.0]	
Both	8 (3.6)	6.0 [4.0–6.8]	
General Experience			
Less than 10 years	152 (69.1)	5.0 [4.0–7.0]	0.345 ^d^
10 years or more	68 (30.9)	5.0 [3.0–7.0]	

^a^Knowledge Score was a range of 0–14, high score reflects more knowledge about cancer pain management)

^b^The *p*-value is bold where it is less than the significance level cut-off of 0.05

^c^Statistical significance of differences calculated using the Mann–Whitney U test

^d^Statistical significance of differences calculated using the Kruskal–Wallis test

### Knowledge of cancer pain management

As shown in Table 2, the rate of correct responses to questions that assess knowledge relating to CPM was calculated; the mean knowledge score was 5.1 with a standard deviation of 2.1. The analysis showed that knowledge deficit was the highest in the question about opioid rescue dose (Opioid rescue dose equals 25% of the basal daily requirement of opioid?) with a correct response rate of 12.3%, and the question about opioid risk of addiction (Opioids as analgesics have a high risk of addiction?) with a correct response rate of 14.1%. A high rate of correct responses was seen in the question about the fastest route for the onset of action of opioids (The IV route for opioid administration has the fastest onset of action?) with a correct response rate of 75.5% and the question about differentiation of certain causes of pain which require specific treatment, with a correct response rate of 72.7%.

**Table 2:** Rate of correct answers about knowledge of cancer pain management

Question^a^	Number of nurses (%)
1. “You should not trust patient’s subjective reports of pain” (F)	104 (47.3)
2. “You should differentiate certain cause of pain which needs specific treatment (i.e. cord compression)” (T)	160 (72.7)
3. “Prescribing a few different types of NSAIDs will increase the analgesic efficacy and decreased adverse effect” (F)	73 (33.2)
4. “Pethidine can be prescribed for chronic cancer pain safely” (F)	67 (30.5)
5. “Opioid analgesics have a high risk of addiction” (F)	31 (14.1)
6. “The effect of immediate release oral opioid can be assessed at 1 h after administration” (T)	83 (37.7)
7. “Opioid analgesics do not have a ceiling effect” (T)	44 (20.0)
8. “Tolerance for opioid-induced sedation develops within a few days” (T)	87 (39.5)
9. “For painful bone metastasis, radiotherapy can alleviate the pain or help to reduce the amount of analgesics” (T)	90 (40.9)
10. “Opioid-induced respiratory suppression is common” (F)	43 (19.5)
11. “Celiac plexus block is effective for treating cancer pain at upper abdomen” (T)	58 (26.4)
12. “Opioid rescue dose equals 25% of the basal daily requirement of opioid” (F)	27 (12.3)
13. “The IV route for opioid administration has the fastest onset of action” (T)	166 (75.5)
14. “Refractory cancer pain rarely occurs with a percent that does not exceed 5% of cancer patients” (F)	94 (42.7)
Mean score, Mean (SD)	5.1 (2.1)

^a^Questions were adapted from Jho et al. [5]

Table 1 shows the association between the knowledge score and the participants’ characteristics. The only factor that showed a significant difference in knowledge score is gender (*p* = 0.001) with a median score [interquartile range] of 6.0 [4.0–7.0] and 5.0 [3.0–6.0] for males and females, respectively.

### Perceived barriers for cancer pain management

Table 3 demonstrates the most important barriers to CPM. Inadequate pain assessment was the most frequent medical staff-related barrier (76.8%). As for patient-related factors, insufficient knowledge of pain control was the most encountered barrier (70.5%). Participants perceived strict regulation of opioids use as the most common health care system-related barrier (69.5%).

**Table 3:** Perceived barriers for cancer pain management (*N* = 220)

Barriers	Number of nurses (%)
Barriers related to medical staff	
Inadequate pain assessment	169 (76.8)
Inadequate experience on pain control	138 (62.7)
Insufficient knowledge of pain control	139 (63.2)
Insufficient communication with patient	146 (66.4)
Reluctance to prescribe opioid	133 (60.5)
Barriers related to patient	
Reluctance to report pain	143 (65.0)
Reluctance to take opioid	124 (56.4)
Insufficient communication with medical staff	140 (63.6)
Financial constraints	141 (64.1)
Insufficient knowledge of pain control	155 (70.5)
Barriers related to the health care system	
Strict regulation of opioids	153 (69.5)
Inadequate staffing	144 (65.5)
Limited stock of different types of opioids	136 (61.8)
Cancer pain management is not considered as important	69 (31.4)
Medication and intervention costs	131 (59.5)

### Pain assessment practices and documentation

Participants rated their training adequacy in CPM as excellent (9.5%), good (42.7%), poor (19.1%) and very poor (28.6%). As shown in Table 4, more than half of the participating nurses would assess the pain on every round (57.7%). Additionally, nurses reported checking all aspects of pain during assessment with severity being the most frequently assessed item. Furthermore, 85.9% of the nurses reported that they document pain assessment.

**Table 4:** Pain assessment and documentation practices (*N* = 220)

Type of practice	Number of nurses (%)
Occasion of pain assessment	
Every round	127 (57.7)
On selected occasions	75 (34.1)
On rare occasions	18 (8.2)
Items checked during pain assessment	
Location	150 (68.2)
Quality	130 (59.1)
Related factor	112 (50.9)
Severity	170 (77.3)
Timing	88 (40.0)
Documentation of pain assessment	189 (85.9)

### Perceptions regarding the most delaying process in opioid administration

Table 5 shows the perceptions regarding the most delaying process in opioid administration. Contacting the physician for the prescription of opioids was cited as the most delaying process during CPM by 56.4% of the participants, followed by obtaining opioids from the pharmacy (15%) and finally the administration of opioids to patients (5%). The causes of delaying processes are difficult to recognize by 23.6% of participants.

**Table 5:** The most common perceptions of delays in cancer pain management (*N* = 220)

Causes of delays	Number of nurses (%)
Administration of opioid to patient	11 (5.0)
Obtaining opioid from pharmacy	33 (15.0)
Contacting physician for prescription of opioid	124 (56.4)
The delaying process is difficult to recognize	52 (23.6)

## Discussions

Our study aimed to assess CPM in terms of knowledge, practices and perceived barriers among Palestinian nurses. Therefore, a questionnaire was developed and distributed with a very good response rate (88%), which can be attributed to many factors including the relatively short duration needed to complete the questionnaire and the nature of the request to complete the survey on the spot.

Our analysis has shown that nurses in Palestine have fair knowledge regarding cancer pain control, with a mean knowledge score of 5.1 out of 14, with knowledge deficits mainly regarding opioid rescue dose calculation, opioids risk of addiction, opioid-induced respiratory suppression, ceiling effect of opioids and utilization of other modalities, such as celiac plexus block. These results were consistent with previous studies which have shown that nurses had the least correct answers for questions asking about the administration of opioids and opioid physical dependency, and have also highlighted knowledge deficits in the physiology and pharmacology of pain [13, 27–30]. Although the main areas of knowledge deficits are consistent with those of the Korean study, the knowledge scores were lower than those achieved by Korean nurses in that study (mean knowledge score among nurses = 9 out of 14, SD = 2.5) [5]. Male nurses scored significantly higher than female nurses.

Moreover, the study participants pointed out important barriers which prevent an adequate control of cancer-related pain, such as the inadequate pain assessment by medical staff, which may be due to inadequate knowledge, an underestimation of the patients’ complaint of pain [31], or inadequate communication and listening to patients [32]. Surprisingly, this shows discordance with the self-assessment of practices in pain assessment and documentation. Close discordance was found in a previous study [6]. Inadequate knowledge of pain control was highlighted as the most commonly encountered patient-related barrier and the strict regulation of opioid use as the most commonly encountered barrier related to the health care system. As barriers to the appropriate treatment of pain can vary based on the standpoint, they are considered from the patient’s, physician’s, or institution’s standpoint, multidisciplinary initiatives should be considered to improve pain management, which may include awareness and education programs in order to change attitudes towards pain treatment [33].

Participants tended to document pain assessment frequently, which is consistent with the previous findings where nurses were reported to have better skills for assessing pain, compared to physicians and pharmacists [5, 30]. The perception of factors that contributed to the delay in the opioid administration process has shown that contacting the physician was the most delaying process which may reflect insufficient coordination between healthcare professionals. Other sources of delay in the process of cancer pain management might exist besides the three processes which were proposed in our question, considering the significant percentage (23.6%) of participants who found difficulty in recognizing the delaying process which may need further investigation.

### Strengths and limitations

To the best of our knowledge, this is the first study to be conducted in Palestine which is concerned with nurses’ knowledge and practices, as well as the perceived barriers relating to the management of cancer pain. The strengths of this study were its relatively large sample size and multicentre design. However, this study has some limitations. Firstly, the study used a convenience sample and thus results may not represent all nurses in Palestine. Secondly, closer monitoring for nursing practices is needed in order to accurately assess the actual practices in CPM. Lastly, this study is cross-sectional, where the causal relationship with the determinants could not be ascertained.

## Conclusions

In conclusion, after analysing the experiences of a sample of nurses who are currently working or previously worked in CPM, we were able to recognise the knowledge deficit with a very low mean knowledge score and the barriers to effective management. On the other hand, the analysis has shown good pain documentation practices among nurses. Those knowledge gaps demonstrate the need for various methods of education on CPM. Educating patients and caregivers about pain management is also important, as the insufficient knowledge of pain control was the most encountered patient-related barrier according to our study. The improvement of coordination and communication between physicians and nurses seems to play a crucial role in CPM, as contacting physicians was cited as the most delaying process in CPM by nurses.

## Additional file

Additional file 1: Study questionnaires. This is the final English and Arabic versions of the questionnaire that was used to obtain data that helps to assess the knowledge and practices of nurses relating to the management of cancer pain, and to determine the barriers to efficient pain control in cancer patients. (DOCX 29 kb)

## Abbreviations

CPM: Cancer pain management
IRB: Institutional review board
SD: Standard deviation
