# Supplementary material for: Nurses’ knowledge, perceived barriers, and practices regarding cancer pain management: a cross-sectional study from Palestine
Source: BMC Med Educ. 2019 May 23;19:167. doi: 10.1186/s12909-019-1613-z (PMC6533684; doi:10.1186/s12909-019-1613-z)
Supplement: Supplementary file 1 — Study questionnaires. This is the final English and Arabic versions of the questionnaire that was used to obtain data that helps to assess the knowledge and practices of nurses relating to the management of cancer pain, and to determine the barriers to efficient pain control in cancer patients. (DOCX 29 kb) [file 12909_2019_1613_MOESM1_ESM.docx]

**Additional file 1: Study questionnaires. This is the final English and Arabic versions of the questionnaire that was used to obtain data that helps to assess the knowledge and practices of nurses relating to the management of cancer pain, and to determine the barriers to efficient pain control in cancer patients.**

**English version***

**Part 1:** **Demographic Data**

| 1. Age ____________ | 2. Male ____ Female ____ | 3. Education (Country) ____________ |
| --- | --- | --- |

4. Type of work:

| 4- Other | 3- Both | 2- private sector | 1- governmental |
| --- | --- | --- | --- |

5. How many years of experience do you have working in a specialized cancer treatment (Include graduate training)?

6. How many years of experience do you have working as a nurse in general?

7. What proportion of your working week is spent attending to patients?

| 1- 33% | 2- 33-66% | 3- 66-100% |
| --- | --- | --- |

8. How frequently do you currently deal with cancer patients who have pain?

| 1- Almost never | 2- Less than once a week | 3- Several times each week |
| --- | --- | --- |
| 4- Daily | 5- More than once each day |  |

9. Please rate adequacy of the training you received in cancer pain management during your training. (Please tick one.)

| ___ Very poor | ___ Poor | ___ Good | ___ Excellent |
| --- | --- | --- | --- |

10. Have you ever experienced pain severe enough to warrant opioid analgesia?

| 2- No | 1- Yes |
| --- | --- |

**Part 2: knowledge of cancer pain management**

For each of the statements below (1-14), please choose (true), (false), or (I don’t know), as best describes the accuracy of that statement, by drawing a sign under the chosen answer.

| Statement | Yes | No | I don’t know |
| --- | --- | --- | --- |
| 1. You should not trust patient’s subjective reports of pain |  |  |  |
| 2. You should differentiate certain cause of pain which needs specific treatment (i.e. cord compression) |  |  |  |
| 3. Prescribing a few different types of NSAIDs will increase the analgesic efficacy and decreased adverse effect |  |  |  |
| 4. Pethidine can be prescribed for chronic cancer pain safely |  |  |  |
| 5. Opioid analgesics have a high risk of addiction |  |  |  |
| 6. The effect of immediate release oral opioid can be assessed at 1 hour after administration |  |  |  |
| 7. Opioid analgesics do not have a ceiling effect |  |  |  |
| 8. Tolerance for opioid-induced sedation develops within a few days |  |  |  |
| 9. For painful bone metastasis, radiotherapy can alleviate the pain or help to reduce the amount of analgesics |  |  |  |
| 10. Opioid-induced respiratory suppression is common |  |  |  |
| 11. Celiac plexus block is effective for treating cancer pain at upper abdomen |  |  |  |
| 12. Opioid rescue dose equals 25 percent of the basal daily requirement of opioid |  |  |  |
| 13. The IV route for opioid administration has the fastest onset of action |  |  |  |
| 14. Refractory cancer pain rarely occurs with a percent that does not exceed 5% of cancer patients |  |  |  |

**Part 3: Perceived barriers for cancer pain management**

In your experience, do the items below stand, as a barrier, in the way of cancer pain management? Please answer by drawing a sign under (yes), (no), or (I don’t know) for each item.

|  | Yes | No | I don’t know |
| --- | --- | --- | --- |
| **Related to medical staff** | | | |
| 1. Inadequate pain assessment |  |  |  |
| 2. Inadequate experience on pain control |  |  |  |
| 3. Insufficient knowledge of pain control |  |  |  |
| 4. Time constrains |  |  |  |
| 5. Reluctance to prescribe opioid |  |  |  |
| 6. Insufficient communication with patient |  |  |  |
| **Patient-related** | | | |
| 1. Reluctance to report pain |  |  |  |
| 2. Reluctance to take opioid |  |  |  |
| 3. Insufficient communication with medical staff |  |  |  |
| 4. Financial constraints |  |  |  |
| 5. Insufficient knowledge of pain control |  |  |  |
| **Related to the health care system** | | | |
| 1. Strict regulation of opioids |  |  |  |
| 2. Inadequate staffing |  |  |  |
| 3. Limited stock of different types of opioids |  |  |  |
| 4. Cancer pain management is not considered as important |  |  |  |
| 5. Medication and intervention costs |  |  |  |

**Part 4: Pain Assessment and documentation practices**

For the following questions, please draw a sign only in front of the selected answer(s), as required, for each question.

1. In your experience, how often do you assess pain in cancer patients? (Please choose one answer only.)

| 1. On every round |  |
| --- | --- |
| 2. On selected occasions |  |
| 3. On rare occasions |  |

2. Which of the following items do you check when assessing the pain? (You can choose more than one answer.)

| 1. Location |  |
| --- | --- |
| 2. Quality |  |
| 3. Related factor |  |
| 4. Severity |  |
| 5. Timing |  |

3. Do you document pain assessment, in the patient’s record, after every encounter with the patient?

| 1. Yes |  |
| --- | --- |
| 2. No |  |

**Part 5: Recognition of delaying processes in cancer pain management**

Which of the following is the most delaying process during cancer pain management in your experience? (Please choose one answer only.)

| 1. Administration of opioid to patient |  |
| --- | --- |
| 2. Obtaining opioid from pharmacy |  |
| 3. Contacting physician for prescription of opioid |  |
| 4. I don’t know |  |

*Most of questions were adapted from Jho HJ, Kim Y, Kong KA, Kim DH, Choi JY, Nam EJ, Choi JY, Koh S, Hwang KO, Baek SK *et al* (2014) **Knowledge, practices, and perceived barriers regarding cancer pain management among physicians and nurses in Korea: a nationwide multicenter survey**. *PLoS One*, **9**(8):e105900.

**Arabic version**

**القسم الأول: معلومات ديموغرافية**

| 3. مكان التعليم (الدولة) __________ | 2. ذكر ____ أنثى ____ | 1. العمر __________ |
| --- | --- | --- |

4**. نوع مكان العمل:**

| 1- حكومي | 2- خاص | 3- كلاهما | 4- غير ذلك: ________ |
| --- | --- | --- | --- |

5. **كم عدد سنوات عملك في مجال علاج مرضى السرطان؟(بما** في ذلك سنوات التدريب)__________ سنة.

6. **كم عدد سنوات عملك في مهنة التمريض بشكل عام؟** __________ سنة.

7. **ما نسبة ما تقضيه/ تقضينه من أسبوع عملك في الاعتناء بالمرضى؟**

| 3- من 66-100% | 2- من 33-66% | 1- أقل من 33% |
| --- | --- | --- |

8. **ما عدد المرات التي تعتني/ تعتنين فيها بمرضى السرطان أسبوعياً في الوقت الحالي؟**

| 3- عدة مرات في الأسبوع | 2- أقل من مرة في الأسبوع | 1- أبدا |
| --- | --- | --- |
|  | 5- أكثر من مرة في اليوم | 4- يوميا |

9. **الرجاء تقييم مستوى التدريب الذي تلقيته في مجال علاج الام مرضى السرطان خلال فترة تدريبك:**

| ___ ممتاز | ___ جيد | ___ ضعيف | ___ ضعيف جدا |
| --- | --- | --- | --- |

10. **هل سبق وعانيت من ألم شديد مما دفعك لاستخدام المسكنات الأفيونية(opioids) ؟**

| 1- نعم | 2- لا |
| --- | --- |

**القسم الثاني: المعرفة بالسيطرة على آلام السرطان**

لكل من الجمل (1–14), يرجى اختيار (صحيح) أو (خطأ) أو (لا أعلم) بما يتناسب مع صحة المعلومة بوضع إشارة عند الإجابة المختارة:

| لا أعلم | خطأ | صحيح | السؤال | الرقم |
| --- | --- | --- | --- | --- |
|  |  |  | يجب أن لا تثق/ تثقي بإخبار المريض عن الألم بشكل ذاتي | 1 |
|  |  |  | يجب تمييز مسببات معينة للألم التي تحتاج إلى علاج خاص بها (مثل انضغاط الحبل الشوكي(Cord Compression) ) | 2 |
|  |  |  | وصف عدة أنواع مختلفة من مضادات الالتهابات غير الستيرويدية (NSAIDS) معا يزيد من الفعالية المسكنة للألم ويقلل من المضاعفات الجانبية | 3 |
|  |  |  | يمكن وصف البيثيدين لعلاج ألم السرطان المزمن بشكل آمن | 4 |
|  |  |  | للمسكنات الأفيونية خطر إدمان عالٍ | 5 |
|  |  |  | يمكن تقييم أثر الانتشار الفوري للمسكنات الأفيونية عن طريق الفم عند الساعة الأولى بعد الإعطاء | 6 |
|  |  |  | المسكنات الأفيونية ليس لها تأثير السقف (ceiling effect) | 7 |
|  |  |  | تتطور ممانعة للتسكين بالمسكنات الأفيونية خلال أيام قليلة من الاستخدام | 8 |
|  |  |  | في حال الانتقالات الثانوية المؤلمة على العظم ((Bone Metastasis, فإن العلاج الإشعاعي قد يخفف من الألم أو يساعد في تقليل كمية المسكنات | 9 |
|  |  |  | يعد تثبيط التنفس الناتج عن المسكنات الأفيونية شائعاً | 10 |
|  |  |  | فعال في علاج ألم السرطان في أعلى البطن (Celiac plexus) سد الظفيرة البطنية | 11 |
|  |  |  | جرعة الإنقاذ الأفيونية تساوي 25٪ من مجموع الجرعة الأفيونية اليومية | 12 |
|  |  |  | إدخال المورفين من خلال الوريد هو أسرع طريق من حيث بدء المفعول | 13 |
|  |  |  | يعتبر ألم السرطان المستعصي نادر الحدوث بنسبة لا تتجاوز 5% من مرضى السرطان | 14 |

**القسم الثالث: العوائق المتصورة للسيطرة على آلام السرطان**

من خلال تجربتك, هل تشكل العوامل التالية عائقاً في السيطرة على الألم لدى مرضى السرطان؟ يرجى الإجابة بوضع إشارة تحت (نعم) أو (لا) أو (لا أعلم) أمام كل من العوامل التالية:

| لا أعلم | لا | نعم |  |
| --- | --- | --- | --- |
| عوائق متعلقة بالطاقم الطبي | | | |
|  |  |  | 1. التقييم غير الكافي للألم |
|  |  |  | 2. الخبرة غير الكافية في السيطرة على الألم |
|  |  |  | 3. المعرفة غير الكافية بالسيطرة على الألم |
|  |  |  | 4. قيود الوقت |
|  |  |  | 5. الممانعة في وصف المسكنات الأفيونية |
|  |  |  | 6. الاتصال غير الكافي مع المريض |
| عوائق متعلقة بالمريض | | | |
|  |  |  | 1. الممانعة في التبليغ عن الألم |
|  |  |  | 2. الممانعة في أخذ المسكنات الأفيونية |
|  |  |  | 3. الاتصال غير كافي مع الطاقم الطبي |
|  |  |  | 4. القيود المالية |
|  |  |  | 5. المعرفة غير الكافية حول السيطرة على الألم |
| عوائق متعلقة بنظام الرعاية الصحية | | | |
|  |  |  | 1. الضوابط الصارمة فيما يتعلق بالمسكنات الأفيونية |
|  |  |  | 2. الطواقم غير الكافية |
|  |  |  | 3. المخزون المحدود من الأنواع المختلفة من المسكنات الأفيونية |
|  |  |  | 4. إدارة آلام السرطان لا يعتبر أمراً مهماً |
|  |  |  | 5. تكاليف الأدوية والتدخلات الطبية |

**القسم الرابع: الممارسة الطبية والتوثيق فيما يتعلق بالسيطرة على آلام السرطان**

لكل من الأسئلة التالية, الرجاء وضع إشارة بجانب الإجابة/ الإجابات المختارة فقط, حسب ما يقتضيه كل سؤال:

1. **في ممارستك الطبية, فإن المناسبات التي تقوم/تقومين فيها بتقييم الألم لدى المريض هي (الرجاء اختيار واحدة فقط):**

|  | 1- في كل مرور/ زيارة للمريض |
| --- | --- |
|  | 2- في مناسبات خاصة (دخول جديد للقسم, عندما يشكو المريض من الألم, عندما يظهر على المريض دلائل الألم) |
|  | 3- في مناسبات نادرة |

2. **أي العناصر التالية تقوم/ تقومين بفحصها أثناء تقييم الألم؟ (يمكن اختيار أكثر من عنصر)**

|  | 1- موقع الألم |
| --- | --- |
|  | 2- طبيعة/ خاصية الألم |
|  | 3- العوامل المصاحبة للألم |
|  | 4- درجة شدة الألم |
|  | 5- توقيت الألم |

3. **هل تقوم/ تقومين بتوثيق البيانات في ملف/ سجل المريض بعد كل عملية تقييم للألم؟**

|  | 1- نعم |
| --- | --- |
|  | 2- لا |

**القسم الخامس: الإدراك لمراحل التأخير في إعطاء المسكنات الأفيونية**

من خلال خبرتك, أي مرحلة من المراحل التالية تعد الأكثر تأخيراً خلال إعطاء المسكنات الأفيونية؟ يرجى وضع إشارة بجانب الإجابة المختارة (الرجاء اختيار واحدة فقط):

|  | 1. ادخال المسكن الأفيوني لجسم المريض |
| --- | --- |
|  | 2. الحصول على المسكن الأفيوني من الصيدلية |
|  | 3. التواصل من الطبيب لوصف المسكن الأفيوني |
|  | 4. لا أعلم |
